# *In vivo* acute toxicity evaluation and *in vitro* molecular mechanism study of antiproliferative activity of a novel indole Schiff base *β*-diiminato manganese^III^ complex in hormone-dependent and triple negative breast cancer cells

**DOI:** 10.7717/peerj.7686

**Published:** 2019-10-07

**Authors:** Reyhaneh Farghadani, Maryam Seifaddinipour, Jayakumar Rajarajeswaran, Mahmood Ameen Abdulla, Najihah Binti Mohd Hashim, Si Lay Khaing, Nur’ain Binti Salehen

**Affiliations:** 1 Department of Molecular Medicine, Faculty of Medicine, University of Malaya, Kuala Lumpur, Malaysia; 2 Institute of Biological Sciences, Faculty of Science, University of Malaya, Kuala Lumpur, Malaysia; 3 Department of Biomedical Science, Faculty of Medicine, University of Malaya, Kuala Lumpur, Malaysia; 4 Department of Pharmacy, Faculty of Medicine, University of Malaya, Kuala Lumpur, Malaysia; 5 Department of Obstetrics and Gynecology, Faculty of Medicine, University of Malaya, Kuala Lumpur, Malaysia

**Keywords:** Indole Schiff base, Apoptosis, Cell cycle, Anti- breast cancer, Caspase, Drug discovery, Manganese complex, Inhibitory activity, Animal study, Antiproliferative

## Abstract

Breast cancer is the most frequently diagnosed cancer among women worldwide. Recently, increasing attention has been paid to the anticancer effects of transition metal complexes of indole Schiff bases. β-diiminato Manganese^III^ complex has shown promising cell cycle arrest and apoptosis induction against MCF-7 and MDA-MB-231 breast cancer cells. In this study, time- and dose- dependent inhibitory activity were evaluated using MTT assay after 48 h and 72 h exposure time. In addition, median effect analysis was conducted according to Chou–Talalay method to investigate whether Mn^III^ complex has synergistic effect in combination with chemotherapeutic drugs on inhibiting breast cancer cell growth. The molecular mechanisms underlying its potent antiproliferative effect was determined through bioluminescent caspase-3/7, -8 and -9 activity assays and quantitative expression analysis of cell cycle- and apoptosis-related genes. Furthermore, safety evaluation of Mn^III^ complex was assessed through the acute oral toxicity test in *in vivo* model. The MTT assay results revealed that it potently reduced the viability of MCF-7 (IC_50_ of 0.63 ± 0.07 µg/mL for 48 h and 0.39 ± 0.08 µg/mL for 72 h) and MDA-MB-231 (1.17 ± 0.06 µg/mL for 48 h, 1.03 ± 0.15 µg/mL for 72 h) cells in dose- and time-dependent manner. Combination treatment also enhanced the cytotoxic effects of doxorubicin but not tamoxifen on inhibiting breast cancer cell growth. The involvement of intrinsic and extrinsic pathway in apoptosis induction was exhibited through the increased activity of caspase-9 and caspase-8, respectively, leading to enhanced downstream executioner caspase-3/7 activity in treated MCF-7 and MDA-MB-231 cells. In addition, gene expression analysis revealed that Mn^III^ complex exerts its antiproliferative effect via up-and down-regulation of p21 and cyclin D1, respectively, along with increased expression of Bax/Bcl-2 ratio, TNF-α, initiator caspase-8 and -10 and effector caspase-3 in MCF-7 and MDA-MB-231 cells. However, the results did not show increased caspase-8 activity in treated MCF-7 cells. Furthermore, *in vivo* acute oral toxicity test revealed no signs of toxicity and mortality in treated animal models compared to the control group. Collectively, the promising inhibitory effect and molecular and mechanistic evidence of antiproliferative activity of Mn^III^ complex and its safety characterization have demonstrated that it may have therapeutic value in breast cancer treatment worthy of further investigation and development.

## Introduction

Cancer is a multi-step process in which cells undergoing metabolic and behavioral changes obtains excessive proliferation and can then invade adjacent parts of the body and/or spread to other organs. Cancer is the second leading cause of death globally and is responsible for an estimated 9.6 million deaths in 2018. That is nearly one in six of all global deaths (Hejmadi, 2009; World Health Organization, 2018). Breast cancer is the second most common cancer overall and the most frequently diagnosed cancer in women worldwide, with over 2 million new cases diagnosed in 2018 representing nearly 25% of all new cancer cases diagnosed among women globally (Bray et al., 2018; Nawaz, 2011). Although currently applied chemotherapeutic drugs play a critical role in breast cancer treatment, important issues regarding chemotherapeutic agents include the emergence of chemoresistance to the drug regimen, which in turn reduces the efficacy of chemotherapy, and disadvantage of widespread adverse side effects such as lowered resistance to infections, weakness, nausea, vomiting and hair loss (Baguley, 2010; De Ruijter et al., 2011; Lu & Chao, 2012). Therefore, discovery and development of new compounds with unique properties has continued to be one of the scientific approaches towards the search for alternative therapeutic agents with reduced toxicity.

Since the discovery of cisplatin, metal-based drugs with extensive clinical applications hold great promise for the development of cancer chemotherapeutic agents. Schiff bases with widespread pharmacological properties attributed to the azomethine C = N functional group can readily form stable complexes with most of the transition metals providing a variety of properties and biological applications. The literature reports have demonstrated increasing interest in metal complexes of Schiff bases as promising agents in the area of cancer drug discovery due to their potent anti-proliferative activity against a variety of cancer cell lines, tumor growth inhibition in animal models and their role in overcoming multidrug resistance (Ganguly et al., 2014; Li et al., 2017; Niu et al., 2016; Rafi et al., 2016; Shanmugam et al., 2018).

Deregulated cell proliferation and suppressed cell death together provide the underlying platform for neoplastic progression of breast cancer. Aberrations in positive regulators, mainly cyclin and cyclin dependent kinase (CDK), and negative regulators including CDK inhibitors of the cell cycle have been reported in breast cancer. Therefore, they represent attractive targets for the intervention of sustained proliferation of carcinoma cells (Ahmad et al., 2012; Elsayed & Sausville, 2001). In addition, it is well-known that apoptosis as a promising target for anticancer therapy is executed through caspases and regulated by the Bcl-2 and TNF receptor family involved in intrinsic and extrinsic pathway, respectively (Pfeffer & Singh, 2018). Caspases, cysteine-aspartic proteases, are classified to initiator (caspase- 8, −10 and −9) or executioner caspases (caspase-3, −6, and −7) based on their position and mechanism of action in apoptotic signaling cascades. Caspase-8 and 10 are involved in extrinsic pathway, while caspase 9 in intrinsic apoptotic pathway. Activation of caspases results in initiation of a protease cascade events leading to various morphological and biochemical changes and rapid cell death. Targeting caspase families, as death-inducing molecules, have also been a powerful therapeutic platform for the development of potent anticancer strategies (Hensley, Mishra & Kyprianou, 2013). Furthermore, death receptors and their ligands have critical role in the extrinsic pathway of apoptosis. Several abnormalities such as down-regulation of the receptor, deficiency of receptor function, and reduced level in the death signals have been reported in impaired death signaling resulting in reduced apoptosis (Ukrainskaya et al., 2017). Besides, BCL-2 family as key regulators of intrinsic apoptosis pathway consist of the anti-apoptotic (e.g., BCL-2, BCL-XL) and the pro-apoptotic (eg, BAX, BIM) proteins which highly regulate mitochondrial outer membrane permeabilization (MOMP) and integrity. However, it is not the absolute quantity but rather the balance between the pro- and anti-apoptotic proteins determines whether apoptosis would be initiated (Montero & Letai, 2018).

In our previous study (Farghadani et al., 2017), we have demonstrated that treatment with indole Schiff base manganese (Mn)^III^ complex significantly inhibited the proliferation of MCF-7 and MDA-MB-231 cells thought the cell cycle arrest at the G_0_/G_1_ phase and apoptosis induction. Henceforth, we have taken a step further and investigated the molecular mechanisms underlying antiproliferative effect of Mn^III^ complex in treated MCF-7 and MDA-MB-231 cells. In addition, a times- and -dose dependent study of its cytotoxicity and its synergistic inhibitory effect in combination with chemotherapeutic drugs including doxorubicin and tamoxifen were evaluated. Furthermore, in order to examine the safety of Mn^III^ complex, *in vivo* animal study was conducted using Sprague Dawley rats.

## Material and Methods

### Cell culture and maintenance

The two human breast cancer cell lines including hormone-dependent MCF-7 and hormone-independent and highly aggressive MDA-MB-231 cell lines were purchased from American Type Culture Collection (ATCC, USA). These MCF-7 and MDA-MB-231 cells were cultured in Dulbecco’s Modified Eagle Medium (DMEM; Sigma) supplemented with 10% fetal bovine serum (FBS; Life Technologies, Carlsbad, CA, USA) and 1% penicillin-streptomycin (Sigma-Aldrich, St. Louis, MO USA). Cells were maintained as monolayer cultures at 37 °C in a humidified atmosphere with 5% CO_2_ and were grown until 70–80% confluence.

### Determination of cell viability

Cell viability was measured by the MTT assay as previously described (Devagi et al., 2017). It is a colorimetric assay based on the reduction of MTT by mitochondrial dehydrogenases of viable cells to a purple formazan product. Briefly, MCF-7 and MDA-MB-231cells were seeded in 96-well cell culture plates at a density of 7 × 10^3^ cells/well. Indole Schiff based β-diiminato ligand (LH_3_) and Mn^III^ complex were dissolved in dimethyl formamide (DMF) (Sigma-Aldrich) to generate the stock solution of 40 mg/mL and further diluted with media to get 100 µg/mL working stock solution for experiments. The maximum concentration of DMF at highest concentration of the compounds was ≤ 0.1% v/v. After overnight growth, MCF-7 and MDA-MB-231 cells were treated with different concentrations of LH_3_ and Mn^III^ complex (0.78, 1.56, 3.12, 6.25, 12.5, 25, and 50 µg/mL) and further incubated for 24 h. In addition, doxorubicin and cisplatin as positive controls, untreated vehicle control and blank with cell-free control were also included. MCF-7 and MDA-MB-231 cells were also treated with a series of Mn^III^ complex concentrations ranging from 0.09 µg/mL to 25 µg/mL for MCF-7 cells and 0.19 µg/mL to 25 µg/mL for MDA-MB-231 cells and incubated for 48 h and 72 h. After exposure time, 50 µl of MTT solution (2 mg/mL in phosphate-buffered saline) was added to each well; the plates were wrapped with aluminium foil to prevent exposure to the light, and further kept in incubator for another 2 h at 37 °C in a 5% CO_2_ humidified atmosphere. Afterwards, the solution was discarded, and 100 mL of DMSO was added to each well to solubilize the crystals. The absorbance was measured at the wavelength of 570 nm using a Tecan infinite M1000Pro microplate reader (Tecan, Männedorf, Switzerland).Each treatment and control was assayed in triplicate in three independent experiments. The IC_50_ (the concentration required for 50% inhibition) was calculated using the GraphPad Prism 5 program (GraphPad Software Inc., San Diego, CA, USA).

### Determination of synergistic effect of Mn^III^ complex in combination with chemotherapeutic drug

In order to evaluate whether Mn^III^ complex could enhance the cytotoxic effects of doxorubicin and tamoxifen on the viability of MCF-7 and MDA-MB-231 cells, the inhibitory effect of their combination was conducted using MTT assay as described earlier. Briefly, after overnight culture, MCF-7 and MDA-MB-231 cells were treated with various doses of doxorubicin and tamoxifen alone or in combination with different concentration of Mn^III^ complex based on their IC_50_value for 24 h. The concentrations used for Mn^III^ complex-doxorubicin combinations included (0.39–0.39, 0.78–0.78, 1.56–1.56, 3.12–3.12, 6.25–6.25 and 12.5–12.5 µg/mL) in both MCF-7 and MDA-MB-231 cells. The concentrations used for Mn^III^ complex-tamoxifen combinations included (0.19–1.56, 0.39–3.12, 0.78–6.25, 1.56–12.5, 3.12–25 and 6.25-50 µg/mL) for MCF-7 cells and (0.39–1.56, 0.78–3.12, 1.56–6.25, 3.12–12.5, 6.25–25 and 12.5-50 µg/mL) for MDA-MB-231 cells. The untreated cells were also included. After 24 h treatment, the cells were further incubated for 2 h with MTT solution and the formation of *purple* formazan *crystals, dissolved in* DMSO, were measured using a Tecan infinite M1000Pro microplate reader (Tecan, Männedorf, Switzerland) at the wavelength of 570 nm. Synergism, additivity, and antagonism were quantified by determining the CI (combination index) calculated by the Chou-Talalay method (Chou, 2010) through CompuSyn software (Combosyn Inc., Paramus, NJ, USA) where CI <1, = 1, and >1 indicate synergism, additive effect, and antagonism, respectively. The linear correlation coefficient (*r*) of the median-effect plot signifies a conformity of the data representing the quality of the data, when *r* = 1, it is considered as perfect. For all experiments, *r* values were between 0.92 and 0.98 indicating good or acceptable data conformity for *in vitro* experiments.

### Determination of Caspase-8, −9, −3∕7 enzymatic activities

The enzymatic activities of caspase-8, −9 and −3∕7 were determined through bioluminescence-based assay using Caspase-Glo^^®^^ 8, 9 and 3/7 Assay kits (Promega Corporation, USA) according to the manufacturer’s protocol. Briefly, MCF-7 and MDA-MB-231 breast cancer cells were seeded at a density of 10^4^ cells per well in a white 96-well microplate and incubated overnight at 37 °C in a humidified atmosphere with 5% CO_2._On the following day, MCF-7 cells were untreated or treated with medium containing 1.5 and 3 µg/mL concentrations of Mn^III^ complex and incubated for 18, and 24 h. In addition, the time point of 12 h was also applied for caspase-8 activity. MDA-MB-231 cells were untreated or treated with 2.5 and 5 µg/mL and further incubated for 18 h and 24 h. However, for executioner caspase 3/7 activity, the 24 h incubation time was considered for treated MCF-7 and MDA-MB-231 cells. After the indicated incubation time, caspase activities were investigated according to the manufacture protocol; equal volume (100 µL/well) of the caspase-Glo reagents was added to each well and further incubated for 45 min at room temperature. Presences of active caspases from apoptotic cells cleave the aminoluciferin-labeled synthetic tetrapeptide. Following this cleavage, a substrate for luciferase is released, resulting in the luciferase reaction and the production of light. The luminescent intensity that is proportional to the amount of caspase enzymatic activity was measured using a Tecan infinite M1000Pro microplate reader (Tecan, Männedorf, Switzerland).

### Real-time polymerase chain reaction (PCR) analysis

The mRNA expression levels of apoptosis- and cell cycle-related genes including Bax, Bcl-2, TNF-*α*, caspase-3, caspase-8, caspase-10, p21, and cyclin D1 were assessed through real-time qPCR analysis. Briefly, MCF-7 and MDA-MB-231 cells were treated with IC_50_concentration of Mn^III^ complex including 1.5 µg/mL and 2.5 µg/mL, respectively, for 24 h. The untreated cells (as control) were also included. After incubation time, the adherent and floating MCF-7 and MDA-MB-231 cells were collected and total mRNA was extracted using a RNeasy plus kit (Qiagen, Valencia, CA, USA) according to manufacturer’s procedure. The quality and integrity of extracted RNA was then determined through the nanodrop spectrophotometer (Thermo Fisher Scientific, Waltham, MA, USA) and 2% agarose gel electrophoresis, respectively. Complementary DNA (cDNA) was then synthesized from the isolated mRNA using a SensiFAST cDNA Synthesis Kit (Bioline, UK) according to the manufacturer’s instructions. Finally, quantitative PCR was carried out on StepOnePlus system (Applied Biosystems) using the SensiFAST™ SYBR^®^ Hi-ROX Kit (Bioline, London, UK) in three replicate. The synthesis was done by an initial denaturation step at 95 °C for 2 min followed by 40 cycles at 95 °C for 5 s, 60 °C for 30 s. The obtained data were then normalized to GAPDH as the reference gene and relative mRNA expression was analyzed by the 2^−(ΔΔCt)^ values (Livak & Schmittgen, 2001). The primer sequences (IDT, Singapore) used in this experiment were shown in Table 1.

**Table 1:** Primer sequences used in real-time quantitative PCR analysis.

Target gene	Forward primer sequence (5′→ 3′)	Reverse primer sequence (5′→ 3′)
p21	GTCACTGTCTTGTACCCTTGTG	CGGCGTTTGGAGTGGTAG
Cyclin D1	GCTGCGAAGTGGAAACCATC	CCTCCTTCTGCACACATTTGAA
Bax	GGACGAACTGGACAGTAACATGG	GCAAAGTAGAAAAGGGCGACAAC
Bcl-2	CTGCACCTGACGCCCTTCACC	CACATGACCCCACCGAACTCAAAGA
TNF- *α*	GCTGTACCTCATCTACTCCCA	GCAATTTCTAGGTGAGGTCTTC
Caspase 3	AGAACTGGACTGTGGCATTGAG	GCTTGTCGGCATACTGTTTCAG
Caspase 8	GTTGTGTGGGGTAATGACAATCT	TCAAAGGTCGTGGTCAAAGCC
Caspase 10	GGACAGTATTCCTGCCGAGG	AATTTCAGCATCCTTGGGACC
GAPDH	GGAGTCCACTGGCGTCTTCAC	GAGGCATTGCTGATGATCTTGAGG

## Acute Toxicity Study

### Animals

Pathogen free female Sprague Dawley (SD) rats (6–8 weeks old) used in the present study was purchased from the Animal House Unit of Faculty of Medicine, University of Malaya (UM). The SD rats were randomly selected and housed in their cages for at least 5 days prior to dosing to allow laboratory condition adaptation. The animals were kept on a 12 h light/dark cycle at 22 ± 3 °C and provided with standard food pellets and tap water. All experimental procedures on the animals were done according to the regulations set by Faculty of Medicine Institutional Animal Care and Use Committee (FOM IACUC).

### Acute toxicity test

In order to evaluate the safety of the complex usage, the acute oral toxicity study was conducted according to the Organization for Economic Co-operation and Development (OECD) guideline for testing of chemicals (OECD, 2001). Since the female rats are slightly more sensitive, female Sprague-Dawley rats (6–8 weeks old) were randomly divided into 3 groups. All experimental procedures were approved by the ethics committee of the Faculty of Medicine, University of Malaya, Malaysia (Ethics Number: 2016-171006/BMS/R/MAA). After overnight fasting with the access to water, the animals were fed orally with a single dosage of 50 and 300 mg/kg of Mn^III^ complex by oral gavage. Before dose administration, the body weight of each animal was determined, and dose was calculated according to body weight. For the vehicle control group, 10% Tween 20 was administered. Feeding was started 3-4 h after dosing and all animals were observed at 30 min, 2, 4, 8, 24, and 48 h up to two weeks to monitor any clinical or toxicological symptoms. After 14 days, all animals were sacrificed, and blood samples were then collected for serum biochemical test. In addition, kidney and liver histological analysis was also performed using Hematoxylin and Eosin (H&E) staining.

### Assessment of kidney and liver functions

The blood samples of SD rat were sent to Clinical Diagnostic Laboratory (CDL) of University Hospital in University of Malaya for renal and liver function tests. As indicators of liver function, several serum parameters including albumin, total bilirubin, alanine aminotransferase, alkaline phosphate, and glutamyl transferase levels were measured. In addition, sodium, potassium, chloride, carbon dioxide, anion gap, urea, and creatinine levels were also measured to assess kidney function.

### Kidney and liver histopathological analysis

The renal and hepatic tissues were placed in cassette and immersed in a 10% formalin solution for 48 h. After that tissue processing was conducted to remove all water from the tissue using automated tissue processor machine. Therefore, tissue was dehydrated through a series of ethanol solutions of increasing concentration to displace the water. Then, in clearing stage, xylene displaced the ethanol in the tissue that this in turn was displaced by molten paraffin wax in the infiltration stage, as the final step in tissue processing. Infiltrated tissues were then embedded into paraffin wax blocks. The tissue blocks were then sectioned at approximately 5 µm thick sections and stained with Hematoxylin and Eosin (H&E). Finally, all sections were observed using light microscope (Leica DM750; Leica, Tokyo, Japan).

### Statistical analysis

The experimental data were expressed as a mean ± standard deviation (SD). A statistical analysis was performed through paired samples *t*-test using SPSS software v.22 to compare the effect between untreated and treated cells. The results were considered statistically significant compared with the control group if *P < 0.05.

## Results

### Effect of Mn^III^ complex treatment on the viability of MCF-7 and MDA-MB-231 breast cancer cells

**Figure 1:**
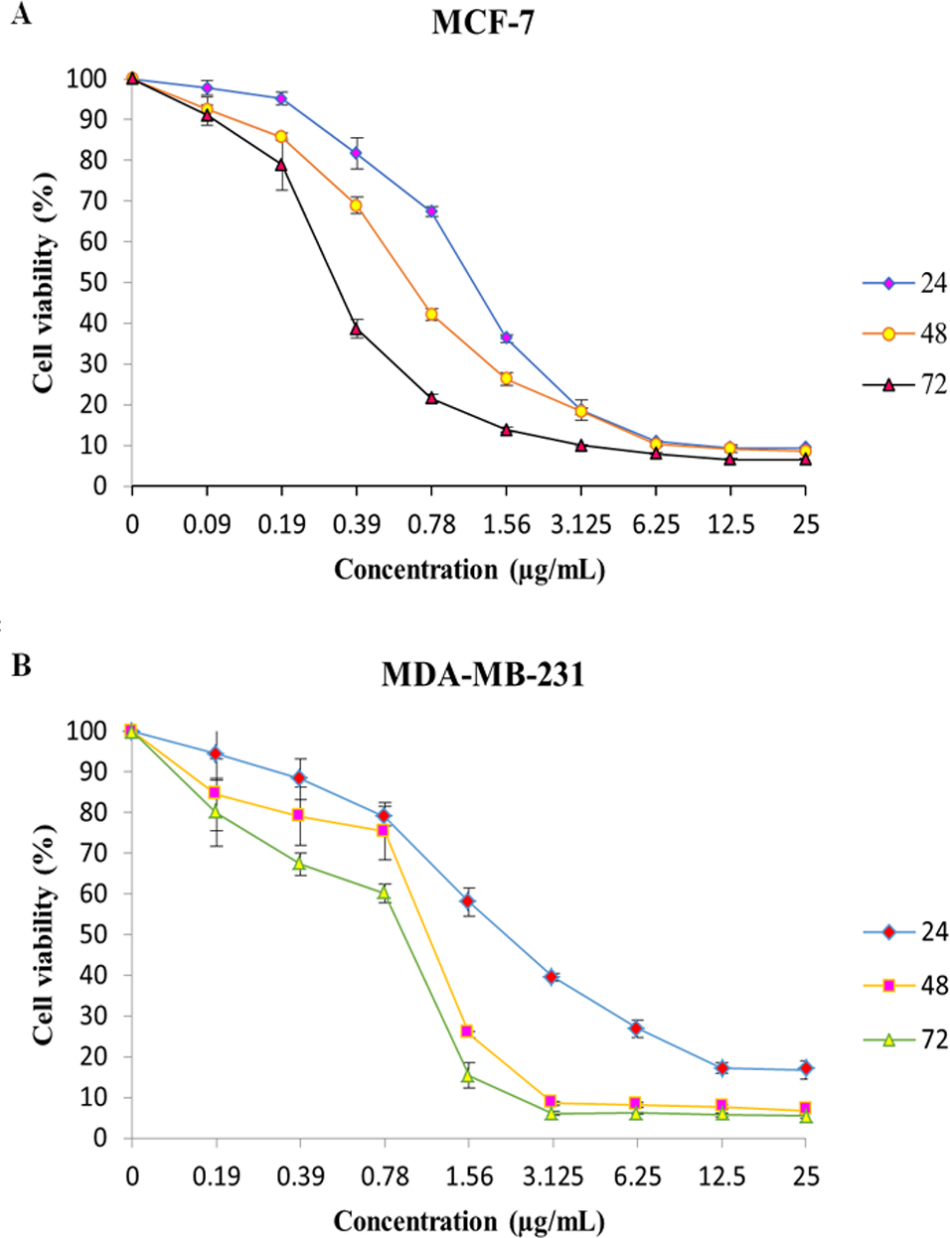
MTT cell viability assay. The breast cancer cells were untreated or treated with Mn^III^ complex at various concentrations for 24, 48 and 72 h, and cell viability was determined using a colorimetric MTT assay. The results revealed dose- and time- dependent decrease in cell viability in both cell lines compared to control group. Here, the control (untreated) group is referred as 100% of viable cells. Data are presented as the mean ± standard deviation.

The effect of Mn^III^ complex and its ligand, indole Schiff-based tetradentate β-diiminato ligand (LH_3_), on viability of MCF-7 and MDA-MB-231 cells have determined using the colorimetric MTT assay. LH_3_ and Mn^III^ complex displayed growth inhibition properties and reduced the MCF-7 and MDA-MB-231 cell viability in a dose-dependent manner with IC_50_ values of 3.51 ± 0.14 and 2.41 ± 0.29 µg/mL for LH_3_ and1.44 ± 0.24 and 2.28 ± 0.38 µg/mL for Mn^III^ complex (Farghadani et al., 2017), respectively, after 24 h treatment. In addition, the IC_50_ value of chemotherapeutic drug, cisplatin, was 8.94 ± 0.66 µg/mL for MCF-7 cells and 6.79 ± 1.22 µg/mL for MDA-MB-231 cells (Table 2). Therefore, these results motivated us to carry out an additional series of experiments in order to investigate the effect of Mn^III^ complex on viability of MCF-7 and MDA-MB-231 cells for longer period of exposure time, 48 h, and 72 h, and establish if Mn^III^ complex was cytotoxic in a time-dependent manner to MCF-7 and MDA-MB-231 cells. As presented in Fig. 1, as exposure period to Mn^III^ complex was prolonged from 24 h to 48 h and 72 h, it exhibited greater inhibitory effect against MCF-7 and MDA-MB-231 cells with an IC_50_ value of (0.63 ± 0.07 µg/mL for 48 h and 0.39 ± 0.08 µg/mL for 72 h) and (1.17 ± 0.06 µg/mL for 48 h, 1.03 ± 0.15 µg/mL for 72 h), respectively.

**Table 2:** The IC_50_ values of LH_3_ and Mn^III^ complex treatment against breast cancer cell lines after 24 h treatment.

**Compound/Drug**	**Cell line**	**IC**_**50**_**(µg/mL)**
**Mn**^III^**complex**	MCF-7	1.44 ± 0.24
	MDA-MB-231	2.28 ± 0.38
**LH**_**3**_	MCF-7	3.51 ± 0.14
	MDA-MB-231	2.41 ± 0.29
**Doxorubicin**	MCF-7	2.55 ± 0.27
	MDA-MB-231	1.84 ± 0.4
**Cisplatin**	MCF-7	8.94 ± 0.66
	MDA-MB-231	6.79 ± 1.22
**Tamoxifen**	MCF-7	9.05 ± 0.19
	MDA-MB-231	10.48 ± 0.45

**Notes.**

Cisplatin, doxorubicin, and tamoxifen were used as positive controls.

### Synergistic inhibitory effects of Mn^III^ complex and doxorubicin combinations on MCF-7 and MDA-MB-231 breast cancer cells

**Figure 2:**
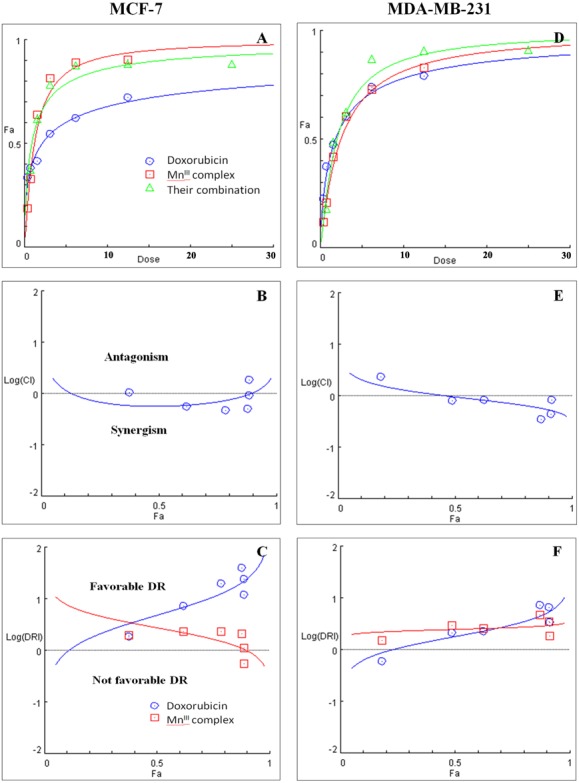
Combination treatment study of Mn^III^ complex and doxorubicin in breast cancer cells. (A) and (D) show the Dose-Fa (fraction affected) curves for Mn^III^ complex, doxorubicin and their combinations in MCF-7 and MDA-MB-231 cells. The combination indexes (CIs), and dose-reduction indexes (DRIs) of the Mn^III^ complex-doxorubicin combinations were calculated by median drug effect analysis using CalcuSyn software as shown in (B) and (E), respectively. The results shown in logarithmic CI/Fa Plot and logarithmic (DRI)/Fa curves revealed that the combination of Mn^III^ complex and doxorubicin exhibited synergistic effect, log CI < 0, in MCF-7 and MDA-MB-231 cells.

**Figure 3:**
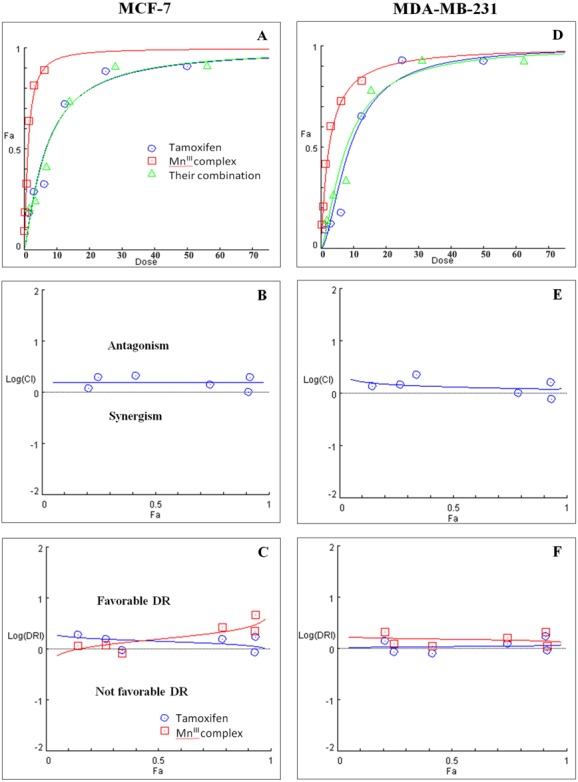
Combination treatment study of Mn^III^ complex and tamoxifen in breast cancer cells. (A) and (D) show the Dose-Fa (fraction affected) curves for Mn^III^ complex, tamoxifen and their combinations in MCF-7 and MDA-MB-231 cells. The combination indexes (CIs), and dose-reduction indexes (DRIs) of Mn^III^ complex- tamoxifen combinations were calculated by median drug effect analysis using CalcuSyn software as shown in (B) and (E), respectively. The results revealed no synergistic interactions of Mn^III^ complex-tamoxifen combinations in either MCF-7 cells or MDA-MB-231 cells except for one dose (log CI < 0).

Initially the effect of doxorubicin and tamoxifen alone on viability of MCF-7 and MDA-MB-231 cells was evaluated using MTT assay to determine the concentration at which cell growth was inhibited by 50%. The results showed that after 24 h of treatment with doxorubicin and tamoxifen, compared to untreated cells, MCF-7 cells reached IC_50_ at 2.55 ± 0.27 µg/mL and 9.05 ± 0.19 µg/mL concentrations and MDA-MB-231 cells reached IC_50_at 1.84 ± 0.4 µg/mL and 10.48 ± 0.45 µg/mL, respectively (Table 2). Subsequently, in order to evaluate the synergistic inhibitory effect of Mn^III^ complex in combination with doxorubicin and tamoxifen, as the most commonly used chemotherapeutic drugs in breast cancer treatment, they were combined based on their corresponding IC_50_ values and then the inhibitory effects of different combinations were compared with single treatments in both cell lines. Taking advantage of the median-effect analysis according to Chou–Talalay methods in calculating combination indexes (CIs), the interaction of the combinations was investigated using CompuSyn software (Chou, 2010). The CIs were calculated to identify the inhibitory effects of the combinations against MCF-7 and MDA-MB-231 cells; the plots of CI versus fraction affected (Fa) were shown in Figs. 2B and 2E. CI <1, = 1, and >1 indicate synergism, additive effect, and antagonism, respectively. The results revealed the synergistic effects of Mn^III^ complex-doxorubicin combinations in both MCF-7 and MDA-MB-231 cells where CI<1 and the combination doses ranged from 1.56–12.5 µg/mL and 1.56–25 µg/mL, respectively (Fig. 2, Table 3). Nevertheless, no synergistic interactions between tamoxifen and Mn^III^ complex were detected in MCF-7 cells as all CI values were detected more than 1 and for MDA-MB-231 cells, the synergism was only observed at the combination treatment of 31.25 µg/mL (Fig. 3, Table 3). In addition, dose-reduction index (DRI) was used to measure the number of reduction folds in the dose produced by the synergistic combination at a given effect level compared with the doses of each drug alone (Chou, 2006). DRI >1, DRI <1 and DRI=1 indicate favorable, not favorable and no dose-reduction. The favorable DRI leads to toxicity reduction in the therapeutic applications. As shown in the Figs. 2C and 2F, after Mn^III^ complex-doxorubicin combination treatment, favorable dose-reduction was detected compared with individual treatment in both cell lines. The DRI values of doxorubicin ranged from 1.84 to 39.93 and 2.14 to 7.39-fold changes in MCF-7 and MDA-MB-231 cells, respectively, whereas those of Mn^III^ complex ranged from 1.13 to 2.35 and 1.5 to 4.74-fold changes, respectively (Table 3).

**Table 3:** Combination treatment study of Mn^III^ complex with doxorubicin and tamoxifen in breast cancer cells.

**A**						**B**				
**Mn**^**III**^**complex –Doxorubicin combinations**						**Mn**^**III**^**complex -Tamoxifen combinations**				
**Cell line**	**tDose**	**CI**	**Effect**	**DRI Dox.**	**DRI Mn**^**III**^**complex**	**tDose**	**CI**	**Effect**	**DRI Tam.**	**DRI Mn**^**III**^**complex**
**MCF-7**	0.78	1.05	Additive	1.84	1.95	1.75	1.19	Antagonistic	1.40	2.09
	1.56	0.56	Synergistic	7.3	2.33	3.51	1.97	Antagonistic	0.84	1.25
	3.12	0.47	Synergistic	19.8	2.35	7.03	2.15	Antagonistic	0.79	1.12
	6.25	0.50	Synergistic	39.93	2.10	14.06	1.42	Antagonistic	1.24	1.62
	12.5	0.92	Synergistic	23.84	1.13	28.12	1.03	Additive	1.76	2.15
	25	1.85	Antagonistic	11.92	0.56	56.25	1.96	Antagonistic	0.92	1.12
**MDA-MB-231**	0.78	2.33	Antagonistic	0.59	1.50	1.95	1.39	Antagonistic	1.92	1.14
	1.56	0.79	Synergistic	2.14	3.00	3.90	1.47	Antagonistic	1.54	1.20
	3.12	0.82	Synergistic	2.29	2.56	7.81	2.26	Antagonistic	0.94	0.82
	6.25	0.34	Synergistic	7.39	4.74	15.62	1.00	Additive	1.56	2.70
	12.5	0.43	Synergistic	6.52	3.53	31.25	0.77	Synergistic	1.76	4.79
	25	0.83	Synergistic	3.43	1.83	62.50	1.62	Antagonistic	0.84	2.25

**Notes.**

Abbreviations tDose total dose CI combination index DRI dose reduction index Dox doxorubicin Tam tamoxifen

### Effect of Mn^III^ complex treatment on caspase-9,-8 and 3/7 enzymatic activity

**Figure 4:**
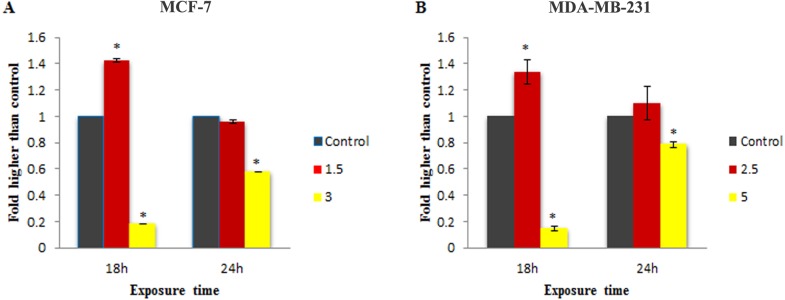
Effect of Mn^III^ complex on caspase-9 enzymatic activities. The MCF-7 (A) and MDA-MB-231(B) breast cancer cells were either untreated or treated with indicated concentrations of the Mn^III^ complex for 18 h and 24 h. The results revealed significant activation of initiator caspase-9 after 18 h of exposure time in treated breast cancer cells compared to control. The results were expressed as the mean ± SD (*n* = 3). ^∗^*P* < 0.05 was considered statistically significant compared with the control group.

To check the involvement of caspase activation in apoptotic cell death induced by LH_3_ and Mn^III^ complex in treated MCF-7 and MDA-MB-231 cells, the enzymatic activity of caspase-9 and caspase-8 as the initiators of the intrinsic and extrinsic apoptosis pathway, respectively, as well as caspase-3/7 as the effector or executioner caspase triggering apoptosis were examined through bioluminescent analysis. The results showed that treatment with IC_50_ concentration of Mn^III^ complex induced a statistically significant increase in caspase-9 activity only after 18 h of exposure time in MCF-7 and MDA-Mb-231 cells, as compared to control. However, as shown in Fig. 4, there was a statistically significant reduction of the caspase-9 activity with increasing concentration of Mn^III^ complex to 3 and 5 µg/mL for MCF-7 and MDA-MB-231 cells, respectively.

**Figure 5:**
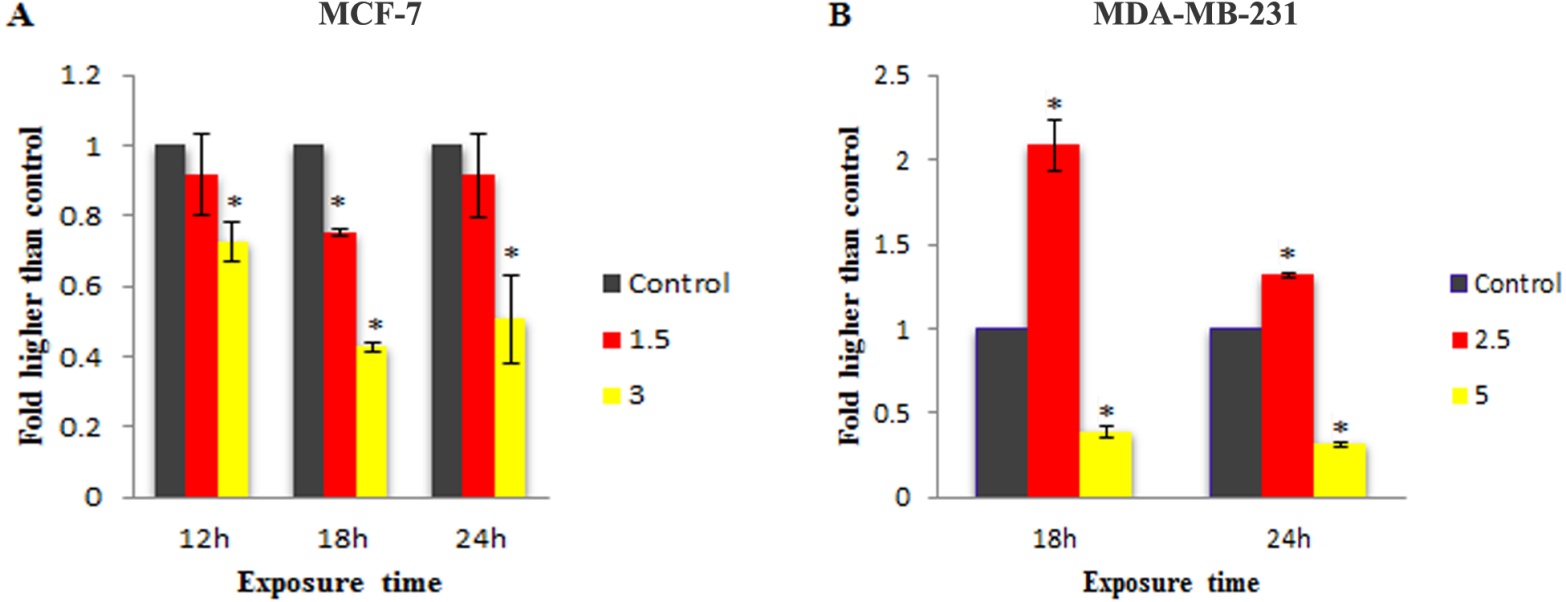
Effect of Mn^III^ complex on caspase-8 enzymatic activities. The MCF-7 (A) and MDA-MB-231 (B) breast cancer cells were either untreated or treated with different concentrations of the Mn^III^ complex for indicated time points. As can be seen here, Mn^III^ complex induced activation of initiator caspases-8 in MDA-MB-231 cells at 18 and 24 h but not in MCF-7 cells, compared to control. The results were expressed as the mean ± SD (*n* = 3).^∗^*P* < 0.05 was considered statistically significant compared with the control group.

Furthermore, as shown in Fig. 5, statistically significant elevation of caspase-8 activity was observed in treated MDA-MB-231 cells with IC_50_ concentration of Mn^III^ complex for 18 h and 24 h, as compared to controls. However, a statistically significant decrease in enzymatic activity of caspase - 8 was observed as the concentration was increased. Since Mn^III^ complex-induced caspase - *8 activation* was not detected in treated MCF-7 cells at 18 and 24 h, we examined caspase-8 activity after 12 h. However, no enhancement in the caspase-8 activity was detected in MCF-7 cells even after 12 h of exposure time.

**Figure 6:**
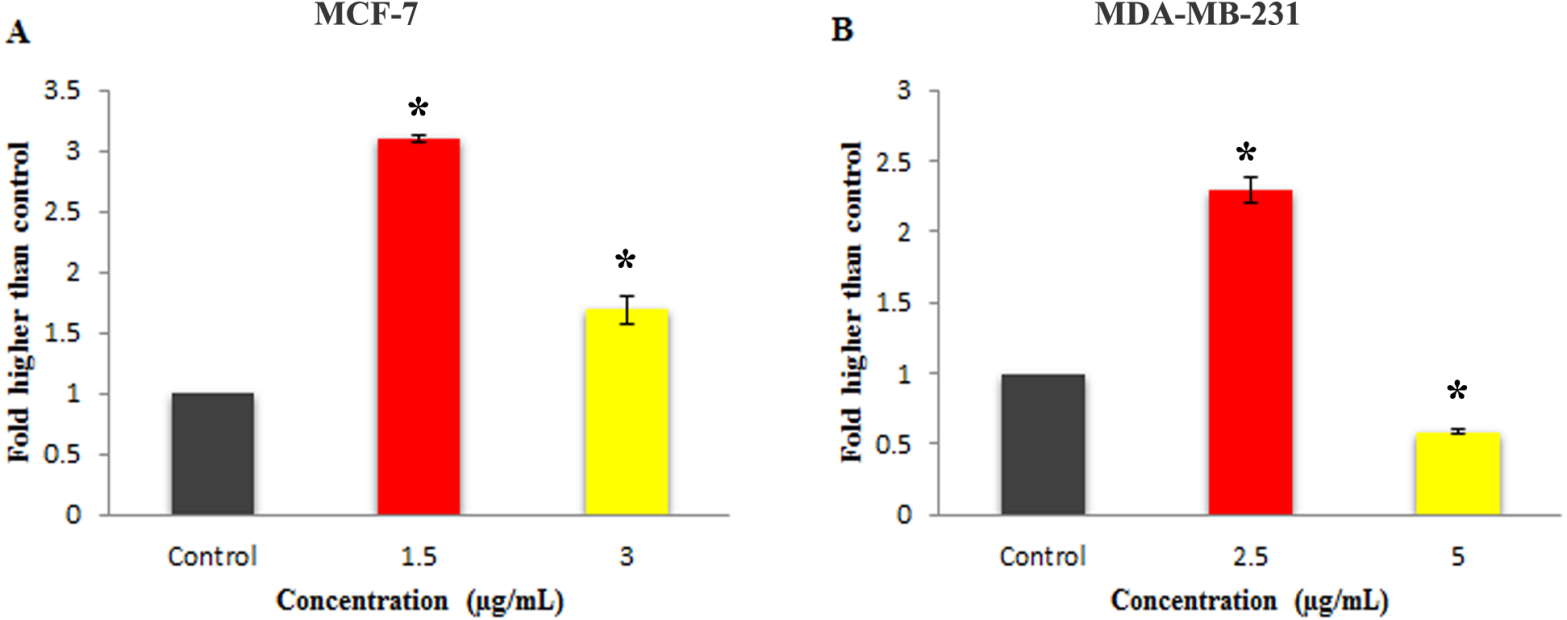
Effect of Mn complex on caspase-3/7 enzymatic activity. The MCF-7 (A) and MDA-MB-231 (B) breast cancer cells were either untreated or treated with indicated concentrations of the Mn^III^ complex and incubated for 24 h. The results have revealed the ability of Mn^III^ complex to induce activation of executioner caspases-3/7 in either MCF-7 or MDA-MB-231 cells compared to control. The results were expressed as the mean ± SD (*n* = 3). ^∗^*P* < 0.05 was considered statistically significant compared with the control group.

Moreover, as shown in Fig. 6, 24 h of treatment with 1.5 and 3 µg/mL concentration of Mn^III^ complex led to a statistically significant elevation in the caspase-3/7 activity in MCF-7 cells as compared to the control. However, statistically significant increase in caspase 3/7 activity was only observed at IC_50_ concentration of Mn^III^ complex in treated MDA-MB-231 cells.

### The effect of Mn^III^ complex on expression of apoptosis- and cell cycle-related genes

**Figure 7:**
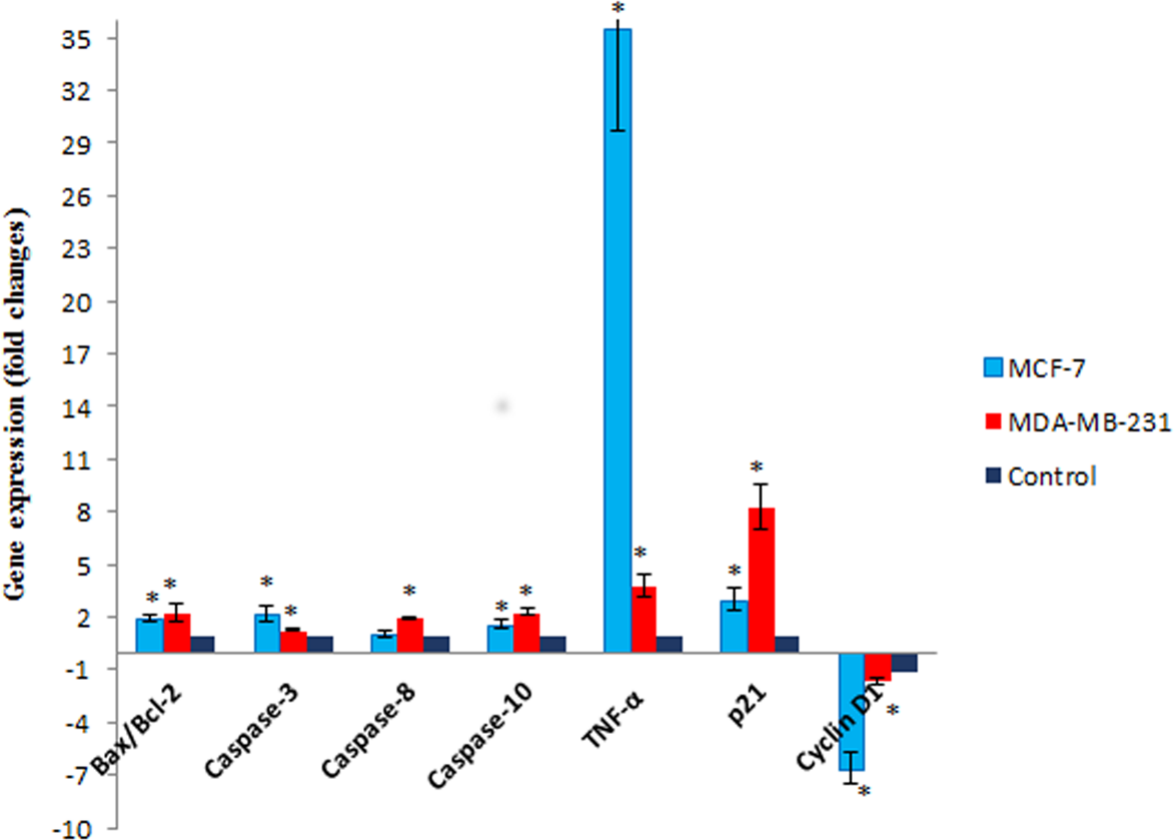
The effect of Mn^III^ complex on expression of apoptosis- and cell cycle-related genes. The MCF-7 and MDA-MB-231 cells were either untreated or treated with Mn complex for 24 h. The mRNA expression levels of indicated genes were determined using real-time PCR. The results indicated an increase in Bax/Bcl-2 ratio and up-regulation of TNF-*α*, caspase-3, −8 and −10. Altered expression of cell cycle-related genes including over expression of p21 along with down-regulation of cyclin D1 were also detected in treated breast cancer cells. The results were expressed as the mean ± SD (*n* = 3). ^∗^*P* < 0.05 was considered statistically significant compared to the control group.

To gain further insight into molecular mechanism underlying antiproliferative activity of Mn^III^ complex, the gene expression analysis of some key molecules known to regulate cell cycle and apoptosis were conducted in MCF-7 and MDA-MB-231 cells after 24 h of exposure. As shown in Fig. 7, Mn^III^ complex caused more than 3- and 8-fold statistically significant increase in p21 gene expression in treated MCF-7 and MDA-MB-231 cells, respectively, compared to control. In addition, the results manifested that Mn^III^ complex significantly down-regulated cyclin D1 expression with ∼6.5-fold in MCF-7 and ∼1.6-fold changes in MDA-MB-231 cells. Besides the cell cycle, the altered expressions of apoptosis regulatory genes were detected. Our data showed that Mn^III^ complex treatment significantly increased Bax/Bcl-2 expression ratio and upregulated the expression of TNF-*α*, initiator caspase-10 and −8 and effector caspase-3 genes in MCF-7 and MDA-MB-231 cells. Based on the result of current study shown in Fig. 7, we found more than 35-fold and 3-fold statistically significant elevation of TNF-*α* gene expression in treated MCF-7 and MDA-MB-231 cells, respectively in both cell lines. Nevertheless, the expression of caspase-8 did not change significantly in MCF-7 cells treated with Mn^III^ complex which was consistent with our bioluminescent caspase-8 activity results.

### Mn^III^ complex induced no significant toxicity in *in vivo* experiment

**Figure 8:**
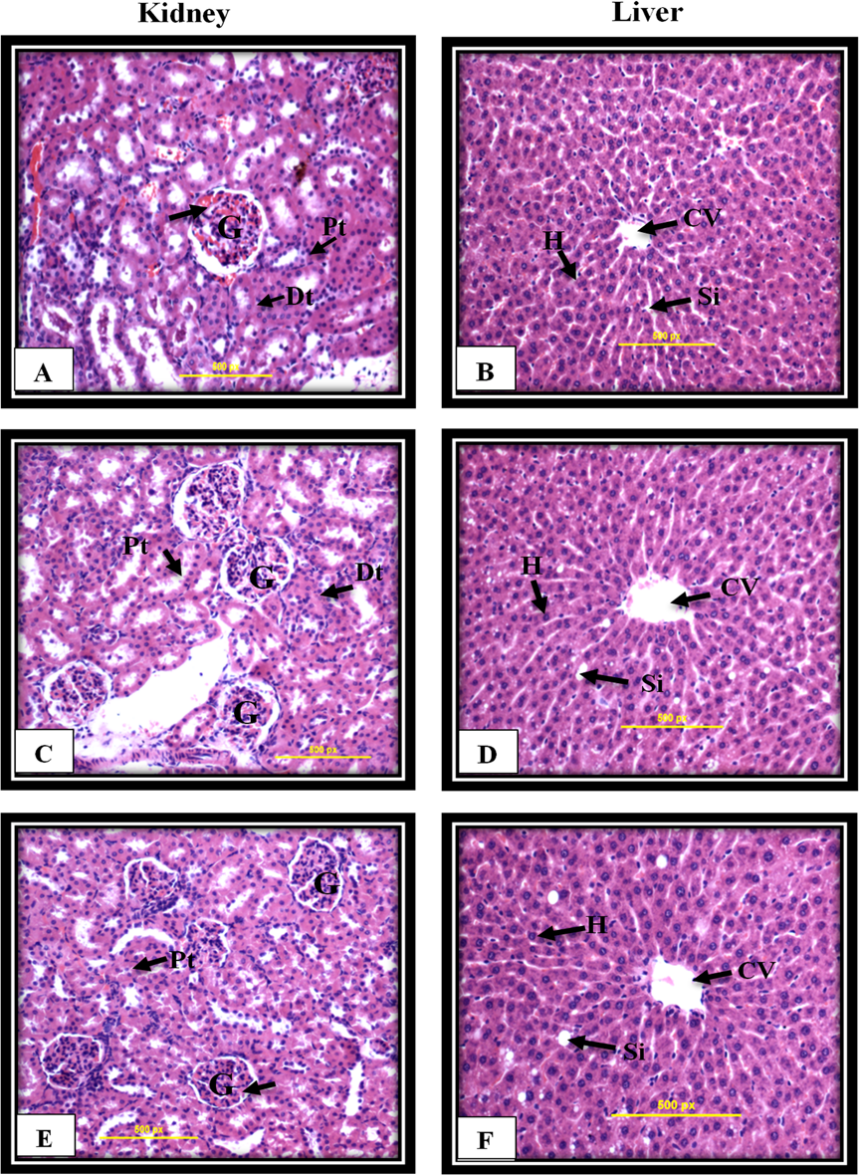
Kidney and liver histological analysis of the acute toxicity experiment. Untreated rats (A and B) received 10% Tween 20 as the vehicle control group. The rats were treated with Mn^III^ complex at 50 mg/kg (C and D) and 300 mg/kg (E and F). No significant differences were observed between the treated and vehicle control group (Hematoxylin and Eosin staining, 20 ×). Glomerulus (G), Distal tubules (Dt), Proximal tubules (Pt), Central vein (CV), Sinusoids (Si), Hepatocytes (H).

The Sprague-Dawley rats were treated once either with 10% Tween 20, as a control group, or Mn^III^ complex at dosage of 50 mg/kg and 300 mg/kg. After 14 days of the experiment, no mortality was observed, and all animals were alive during the treatment period. There were no changes in body weight or gross appearance on the experimental animals and neither physical abnormalities nor behavior changes were observed during the treatment period. Furthermore, histopathological analysis of vital organs including kidney and liver shown in Fig. 8 indicated that there were no hepatotoxic or nephrotoxic effects in the treated rats as compared to control group. Furthermore, biochemical analysis of kidney and liver function parameters, shown in Tables 4 and 5, indicated that there were no statistically significant changes representing no hepatotoxic or nephrotoxic effects in the treated rats as compared to control group.

**Table 4:** Effect of Mn^III^ complex on kidney biochemical parameters in rats. The results were expressed as the mean ± SD (*n* = 3). There were no statistically significant differences between treated groups compared to control group. ^∗^*P* < 0.05 was considered statistically significant compared to the control group.

**Groups**	**Vehicle control**	**Mn**^III^**complex (50 mg/kg)**	**Mn**^III^**complex (300 mg/kg)**
Sodium (mmol/L)	141.3 ± 1.52	140 ± 1	141 ± 1
Potassium (mmol/L)	4.73 ± 0.20	4.9 ± 0.34	4.75 ± 0.21
Chloride (mmol/L)	100.6 ± 1.52	100.3 ± 1.15	102 ± 0
CO_**2**_ (mmol/L)	26.3 ± 0.57	27 ± 2.64	23.33 ± 2.08
Anion gap (mmol/L)	18.6 ± 1.52	17.66 ± 1.15	19 ± 1.41
Urea (mmol/L)	5.46 ± 0.51	5.6 ± 1.01	6.15 ± 0.77
Creatinine (mmol/L)	30.3 ± 0.57	27.33 ± 5.13	32.33 ± 2.30

**Table 5:** Effect of Mn^III^ complex on liver biochemical parameters in rats. The results were expressed as the mean ± SD (*n* = 3). There were no statistically significant differences between treated groups compared to control group. ^∗^*P* < 0.05 was considered statistically significant compared to the control group.

**Groups**	**Vehicle control**	**Mn**^III^**complex (50 mg/kg)**	**Mn**^III^**complex (300 mg/kg)**
Albumin (g/L)	37.33 ± 1.15	36.33 ± 0.57	35.33 ± 0.5
Total bilirubin (µmol/L)	<2	<2	<2
Alkaline phosphate (IU/L)	155.66 ± 40.4	116 ± 15.50	93.66 ± 20.74
Alanine minotransferase (IU/L)	48.33 ± 12.74	36 ± 2.64	49 ± 2.64
Glutamyle Transferase (IU/L)	<6	<6	<6

## Discussion

In this study antiproliferative activity of a novel indole Schiff-based Mn^III^ complex were evaluated against human breast cancer cell lines that provide an excellent platform for breast cancer research in tumour progression and treatment. The MCF-7 and MDA-MB-231 cell lines have been considered as a suitable model for drug discovery in breast cancer study. The MCF-7 cell line as a widely studied model for hormone-dependent human breast cancer has functional estrogen receptors and is often chemotherapy responsive. However, MDA-MB-231 cell line characterized by the lack of expression of estrogen receptor (ER −), progesterone receptor (PR −), and human epidermal growth factor receptor 2 (HER2 −) is an extremely aggressive, hormone-independent and often resistant to chemotherapeutic drug, making it an ideal model for triple-negative breast cancer research (Aka & Lin, 2012; Holliday & Speirs, 2011). The obtained MTT results have indicated that Mn^III^ complex potently reduced the cell viability of MCF-7 and MDA-MB-231 cells in a dose- and time-dependent manner and was more cytotoxic compared to other chemotherapeutic drugs including cisplatin, doxorubicin and tamoxifen. However, there was no significant difference between doxorubicin- and Mn^III^ complex- MDA-MB-231 treated cells, indicating a comparable anti-tumor efficiency between them. Additionally, in comparison to its ligand, an indole Schiff-based tetradentate *β*-diiminato ligand (LH_3_), Mn^III^ complex induced greater inhibitory effect against MCF-7 and MDA-MB-231 cells especially for hormone-dependent MCF-7 cells after 24 h treatment. These data support previous studies showing the metal ions may modify and enhance the biological activity of ligand (Al-Sha’alan, 2007; Halli & Sumathi, 2017; Mishra, Mishra & Pandey, 2011). Furthermore, the possible modulatory effect of Mn^III^ complex on the inhibitory activity of chemotherapeutic drugs was assessed through MTT assay. The aim was to explore the interaction and combination effect between Mn^III^ complex and two widely used drugs in cancer chemotherapy, doxorubicin, and tamoxifen, against MCF-7 and MDA-MB-231 breast cancer cells. Although doxorubicin interacts with DNA by intercalation inhibiting cancer cell growth, tamoxifen is a selective estrogen receptor modulator making them suitable for combination study due to their distinct mechanism of actions (Saueressig et al., 2018; Thorn et al., 2011; Vethakanraj & Kumar, 2017). The combination treatment results indicated the broad spectrum of synergistic effect of Mn^III^ complex-doxorubicin combinations in inhibiting breast cancer cell growth, but the exact mechanism involved remains to be elucidated. However, such synergism was not detected in Mn^III^ complex-tamoxifen combinations.

Apoptosis is a complex activity which is mediated by numerous molecules and is categorized into caspase-dependent or caspase-independent mechanisms (Constantinou, Papas & Constantinou, 2009). To figure out the involvement of caspase cascade events underlying apoptosis process, MCF-7 and MDA-MB-231 cells were stained with aminoluciferin-labeled substrate of caspases and determined the enzymatic activity of caspase-3/7, −8, −9 by quantifying the luminescence intensities in indicated time points and concentrations. As the potential of Mn^III^ complex to induce the ROS generation has been proved in our previous study (Farghadani et al., 2017) and in agreement with other studies revealing that caspase-9 is activated as a downstream target of ROS elevation which is connected with mitochondrial intrinsic pathway (Gao et al., 2015; Hajrezaie et al., 2015; Zheng et al., 2018), incubation with Mn^III^ complex led to enhanced caspase-9 activity in treated MCF-7 and MDA-MB-231 cells representing the involvement of intrinsic pathway in apoptosis induction in both cell lines. Furthermore, there is an evidence that ROS may also cause cellular apoptosis mediated by death receptors which is activated by caspase-8, as an early apoptotic marker and initiator of the extrinsic apoptosis pathway (Redza-Dutordoir & Averill-Bates, 2016). Mn^III^ complex led to enhanced activity of caspases-8 in treated MDA-MB-231 cells but not in MCF-7 cells representing the involvement of extrinsic cell death in apoptosis induction by Mn^III^ complex treatment. Although caspases-8 and -9 initiate the apoptosis signal, once activated, caspase 3/7 as the executioner caspase in apoptotic signaling pathway promote initiation of a protease cascade events by targeting a number of critical cellular proteins such as structural and DNA repair proteins which lead to irreversible morphological and biochemical changes (Kitazumi & Tsukahara, 2011; Li & Yuan, 2008). Therefore, the activity of executioner caspase 3/7 was investigated in Mn^III^ complex -treated MCF-7 and MDA-MB-231 cells. The analysis of caspase-3/7 activity confirmed that apoptotic morphological changes such as membrane blebbing ,chromatin condensation and phospholipid externalization which were previously reported (Farghadani et al., 2017) in Mn^III^ complex-treated MCF-7 and MDA-MB-231 cells were associated with enhanced caspases 3/7 activity (Doonan & Cotter, 2008; Segawa & Nagata, 2015). The expression and abundance of each caspase in a particular cell line will vary and activation and cleavage of caspases in the cascade differ in response to a variety of death stimuli and will change over time. Moreover, caspase activity is short-lived and gradually declines as the cells undergo secondary necrosis and rupture that cause discharging cytoplasmic constituents into the culture medium (Morgan & Thorburn, 2001; Parrish, Freel & Kornbluth, 2013). These are the reasons explaining the differential responses of caspase activity detected in Mn^III^ complex-treated MCF-7 and MDA-MB-231 cells.

To further investigate the underlying genes responsible for the anticancer potential of Mn^III^ complex treatment against MCF-7 and MDA-MB-231 cells, RT-qPCR analysis was performed to study the gene expression of the genes involved in apoptosis and cell cycle induction. Aberrations in many negative and positive regulators of the cell cycle such as defective function of cyclin-dependent kinase inhibitors (CDKI) and up-regulation of cyclins have been reported in breast cancer. There is a strong evidence that functional loss of CDKI such as p21, a well-characterized inhibitor of the cell cycle, not only cause to breast cancer progression, but also play an important role in drug-resistance effect during treatment (Abukhdeir & Park, 2008; Ahmad et al., 2012). Results from this study revealed that Mn^III^ complex exposure lead to significant increase of p21 as an inhibitor for cell growth in both breast cancer cell lines. Doxorubicin and tamoxifen, the most widely used antitumor drugs used for breast cancer treatment, induce their inhibitory effect through increased expression of p21 as well (Cariou et al., 2000; Tarasewicz et al., 2014). Additionally, down regulation of cyclin D1 gene expression was detected in Mn^III^ complex-treated cells. Among different cyclins, cyclin D1 with oncogenic growth-promoting properties is probably the most extensively studied in breast tumors that its mRNA overexpression has been reported in more than 50% of breast cancer cases. Having CDK dependent and independent functions, cyclin D1 may cause to maturation and differentiation of tumor cells and its over expression is expected to be associated with poor prognosis as well (Ishii et al., 2006; Mohammadizadeh et al., 2013). Therefore, induction of cell cycle arrest through up-regulation of CDKIs to re-sensitize cancer cells and down-regulation of cyclins and cyclin-dependent kinases (CDKs) is an important mechanism of cancer therapeutic agents in breast cancer cells (Musgrove et al., 2011; Parveen et al., 2016). Collectively, Mn^III^ complex exerted its cytostatic effects on MCF-7 and MDA-MB-231 cells in a similar way through up-regulation of inhibitory p21 and down-regulation of cyclin D1, as an inhibitor and promoting factor for cell growth, respectively, which contribute to G1 cell cycle arrest in both treated MCF-7 and MDA-MB-231 cells.

Besides the cell cycle, the mRNA levels of apoptosis regulatory genes were investigated to obtain a better molecular insight into the apoptosis mechanism. Accumulating evidences show that Bcl-2 family members are the major regulators of mitochondrial apoptotic cell death which include pro-apoptotic proteins (e.g., Bax, Bak, Bad), and the anti-apoptotic proteins (e.g., Bcl-2, Bcl-XL), regulating the release of proteins from mitochondrial intermembrane space into the cytosol. It is not the absolute quantity but rather the balance between the pro- and anti-apoptotic proteins determines whether mitochondria-mediated apoptosis would be initiated (Montero & Letai, 2018; Wang & Youle, 2009). Our data showed that Mn^III^ complex treatment significantly increased Bax/Bcl-2 expression ratio in both cell lines, implying the relevance of Bcl-2 family proteins for intrinsic apoptosis induction. These proteins control cell death by regulating the mitochondrial outer membrane permeabilization (MOMP). The presence of Bcl-2 protein in mitochondria blocks cell death by inhibiting apoptosis-associated release of cytochrome c from the mitochondria, while effector pro-apoptotic protein, Bax, oligomerize in the mitochondrial outer membrane (MOM) resulting in protein-lined channels or pore formation which cause an increase of MOMP and the loss of MMP, which was detected in our previous experiment (Farghadani et al., 2017). This permeabilization allows the release of cytochrome c into the cytosol followed by formation of apoptosome, which activates caspase 9 and triggers initiation of intrinsic apoptotic pathway (Montero & Letai, 2018; Ola, Nawaz & Ahsan, 2011; Williams & Cook, 2015). Accordingly, an elevated Bax/Bcl-2 expression ratio upon treatment with Mn^III^ complex caused to MMP disruption, which facilitated cytochrome c release and activation of caspase cascade event as evidenced by up-regulation of caspase-3 gene expression in treated MCF-7 and MDA-MB-231 cells. Furthermore, it is well known that the activation of tumor necrosis factor (TNF) family death receptors mediate extrinsic pathway of apoptosis through their death domain that is critical for apoptosis signal transduction. Amongst notable ligands and their corresponding death receptors including FasL/FasR, TNF-*α*/TNFR1, Apo3L/DR3, Apo2L/DR4, and Apo2L/DR5, TNF-*α* which promotes apoptosis through binding to the TNF-receptor 1 is the characteristic model in the extrinsic apoptotic pathway (Elmore, 2007; Schmitz, Kirchhoff & Krammer, 2000). The result of current study represent a major role TNF-*α* in extrinsic apoptosis induction of Mn^III^ complex in MCF-7 and MDA-MB-231 cells. The binding of TNF-*α* to its death receptor cause oligomerization of the TNFR1, recruitment of TNF receptor associated death domain (TRADD) and initiator caspases (e.g., procaspase-8 or -10). Subsequently, these death-inducing signaling complexes promotes the oligomerization of procaspase-8 and -10 followed by auto-activation through self-cleavage which in turn activates downstream effector caspases-3/7 (Dickens et al., 2012; Lavrik, Golks & Krammer, 2005). Accordingly, our results revealed that treatment with Mn^III^ complex caused statistically significant over expression of initiator caspase-8, -10 and effector caspase-3. Although the caspase-8 is the key initiator caspase involved in the activation of extrinsic pathway, it has been reported that apoptosis signaling by death receptors involves not only caspase-8 but also caspase-10 and each caspase can initiate apoptosis independently. Therefore, both caspases may have equal importance in apoptosis initiation (Backman, Eriksson & Danielson, 2013; Kischkel et al., 2001; Meng et al., 2010; Wang et al., 2001). The gene expression analysis of this study showing no statistically significant activation of caspase-8 in Mn^III^ complex-treated MCF-7 cells brought the idea that caspase-10 was recruited by death receptor and might initiate apoptosis independently. In addition, some studies have demonstrated that the extrinsic pathway can crosstalk to the intrinsic pathway through the caspase-8,10-mediated cleavage of Bid, as the pro-apoptotic member of *Bcl - 2* family protein. Activated has the ability to induce MOMP and provides the link between death receptor stimulation and mitochondrial apoptotic events as well (Garrido et al., 2006; Kantari & Walczak, 2011). Together, we provide molecular and mechanistic evidence that Mn^III^ complex activated both intrinsic and extrinsic signaling pathway of apoptosis in treated MCF-7 and MDA-MB-231 cells through statistically significant increased Bax/Bcl-2 expression ratio and TNF-*α* along with enhanced expression of relevant genes in the caspase family that were consistent with our previous findings.

Acute oral toxicity study is the initial step in the assessment and evaluation of the toxic characteristics of all compounds using animal model which enable compound to be ranked for classification purposes and hazard assessment and is essential for drug development process. Using rodents as models in safety evaluations of compounds is currently required in both the pharmaceutical and chemical international guidelines. (Akhila, Shyamjith & Alwar, 2007; Parasuraman, 2011). According to OECD guidelines, the preferred rodent species is the female rats as they are more sensitive (OECD, 2001). In the present study, *in vivo* acute oral toxicity test of Mn^III^ complex on Sprague-Dawley female rats was conducted according to the OECD guidelines for the testing of chemicals (OECD, 2001). The *in vivo* acute oral toxicity test on Sprague-Dawley rats revealed that there was no mortality on the experimental animals during the assay in treated animal models compared to the control group confirming the safety characterization of Mn^III^ complex usage. Similarly, several other studies showed that the Schiff base derivatives were safe, and no related toxicity was detected (Faraj et al., 2014b; Hajrezaie et al., 2014; Zahedifard et al., 2015).

**Figure 9:**
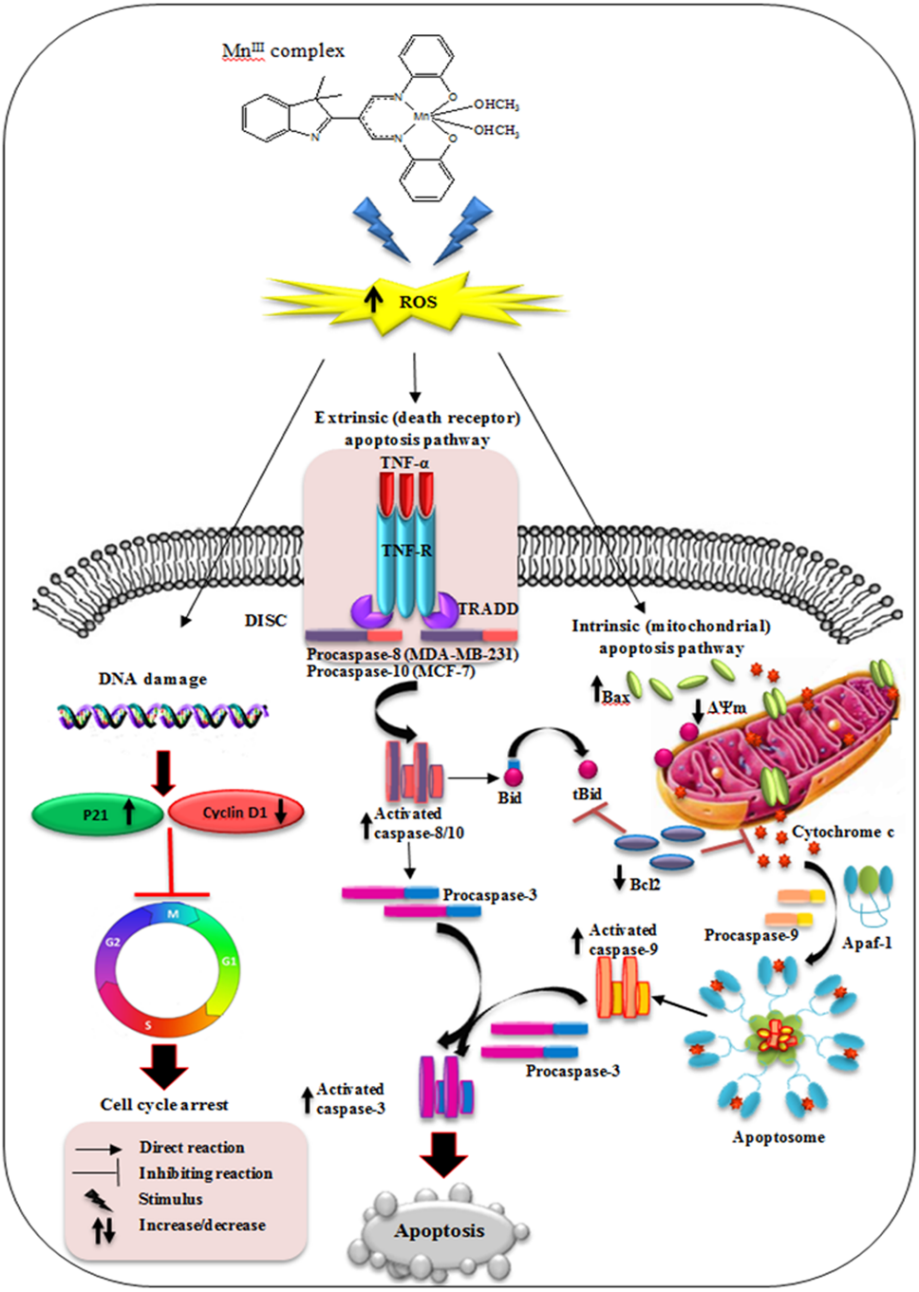
Schematic diagram of mechanism of Mn^III^ complex anti-breast cancer activity.

## Conclusion

In conclusion, our results have shown that Mn^III^ complex exerts potential inhibitory effect on breast cancer cell growth in a dose- and time- dependent manner and enhances the cytotoxic effect of doxorubicin on MCF-7 and MDA-MB-231 cells. The results revealed that Mn^III^ complex induced cell growth arrest by modulating the expression of cell cycle inhibitor and promoting factors including p21 and cyclin D1, respectively, on MCF-7 and MDA-MB-231 cells. Furthermore, Mn^III^ complex-induced apoptosis in MCF-7 and MDA-MB-231 cells involved both the extrinsic and intrinsic signaling pathway as shown in Fig. 9. This complex affects the intrinsic apoptotic pathway by modulating expression level of anti- and pro-apoptotic proteins of Bcl-2 family and activating initiator caspase-9 as well as affects extrinsic apoptotic pathway by increased TNF-*α* gene expression and activation of initiator caspase-8 and-10 in MDA-MB-231 and caspase-10 in MCF-7 cells. Therefore, initiator caspases (caspas- 8, -9 and-10) initiate the apoptosis signal and activate executioner caspase-3/7, which is induced by Mn^III^ complex treatment, promote caspase cascade events leading to the cell death in both MCF-7 and MDA-MB-231 cells. Moreover, acute toxicity results in rats did not show any biochemical or histopathological signs of toxicity. Hence, the promising inhibitory effect and molecular and mechanistic evidence of potent antiproliferative activity of Mn^III^ complex and its safety characterization have demonstrated that it may have therapeutic value in breast cancer treatment worthy of further investigation and development. Furthermore, this class of compounds as a potential lead molecule with promising antiproliferative activity can be focused further in medicinal chemistry to design, synthesis and establish novel and more effective anticancer agents.

##  Supplemental Information

10.7717/peerj.7686/supp-1 Supplemental Information 1 Raw data

10.7717/peerj.7686/supp-2 Supplemental Information 2 Raw data of kidney and liver biochemical parameters

10.7717/peerj.7686/supp-3 Supplemental Information 3 Raw data of Melting curve

## Additional Information and Declarations

### Data Availability

The following information was supplied regarding data availability: Raw data is available as a Supplemental File.
