# Supplementary material for: In vivo acute toxicity evaluation and in vitro molecular mechanism study of antiproliferative activity of a novel indole Schiff base β-diiminato manganeseIII complex in hormone-dependent and triple negative breast cancer cells
Source: PeerJ. 2019 Oct 7;7:e7686. doi: 10.7717/peerj.7686 (PMC6786247; doi:10.7717/peerj.7686)
Supplement: Supplemental Information 2 [file peerj-07-7686-s002.docx]

**Table 4: Effect of Mn^ІІІ^ complex on kidney biochemical parameters in rats.**

| **Groups** | **Sodium (mmol/L)** | **Potassium (mmol/L)** | **Chloride (mmol/L)** | **CO_2_ (mmol/L)** | **Anion gap (mmol/L)** | **Urea (mmol/L)** | **Creatinine (mmol/L)** |
| --- | --- | --- | --- | --- | --- | --- | --- |
| VC1 | 140 | 4.9 | 99 | 27 | 19 | 5.6 | 31 |
| VC2 | 141 | 4.5 | 102 | 26 | 17 | 5.9 | 30 |
| VC3 | 143 | 4.8 | 101 | 26 | 20 | 4.9 | 30 |
| Mean | 141.33 | 4.73 | 100.66 | 26.33 | 18.66 | 5.46 | 30.33 |
| SD | 1.52 | 0.20 | 1.52 | 0.57 | 1.52 | 0.51 | 0.57 |
| L1 | 141 | 5.1 | 101 | 28 | 17 | 6.7 | 33 |
| L2 | 140 | 4.5 | 99 | 29 | 17 | 4.7 | 23 |
| L3 | 139 | 5.1 | 101 | 24 | 19 | 5.4 | 26 |
| Mean | 140 | 4.9 | 100.33 | 27 | 17.66 | 5.6 | 27.33 |
| SD | 1.00 | 0.34 | 1.15 | 2.64 | 1.15 | 1.01 | 5.13 |
| H1 | 140 | 4.6 | 102 | 24 | 18 | 6.7 | 31 |
| H2 | 141 | 5.3 | 102 | 21 | 23 | 9.3 | 31 |
| H3 | 142 | 4.9 | 102 | 25 | 20 | 5.6 | 35 |
| Mean | 141 | 4.75 | 102 | 23.33 | 19 | 6.15 | 32.33 |
| SD | 1.00 | 0.21 | 0.00 | 2.08 | 1.41 | 0.77 | 2.30 |

VC: Vehicle control (n=3), L:low dose of Mn^ІІІ^ complex (50 mg/kg)( n=3), H: high dose of Mn^ІІІ^ complex (300 mg/kg) (n=3).

**Table 5: Effect of Mn^ІІІ^ complex on liver biochemical parameters in rats.**

| **Groups** | **Albumin (g/L)** | **Total bilirubin (μmol/L)** | **Alkaline phosphate (IU/L)** | **Alanine minotransferase (IU/L)** | **Glutamyle Transferase (IU/L)** |
| --- | --- | --- | --- | --- | --- |
| VC1 | 38 | <2 | 181 | 42 | <6 |
| VC2 | 36 | <2 | **109** | **63** | <6 |
| VC3 | 38 | <2 | 177 | 40 | <6 |
| Mean | 37.33 | <2 | 155.66 | 48.33 | <6 |
| SD | 1.15 | - | 40.46 | 12.74 | - |
| L1 | 36 | <2 | 99 | 39 | <6 |
| L2 | 36 | <2 | 128 | 34 | <6 |
| L3 | 37 | <2 | 123 | 35 | <6 |
| Mean | 36.33 | <2 | 116.66 | 36 | <6 |
| SD | 0.57 | - | 15.50 | 2.64 | - |
| H1 | 35 | <2 | 116 | 51 | <6 |
| H2 | 35 | <2 | 75 | 50 | <6 |
| H3 | 36 | <2 | 90 | 46 | <6 |
| Mean | 35.33 | <2 | 93.66 | 49 | <6 |
| SD | 0.57 | - | 20.74 | 2.64 | - |

VC: Vehicle control (n=3), L: low dose of Mn^ІІІ^ complex (50 mg/kg) ( n=3), H: high dose of Mn^ІІІ^ complex (300 mg/kg) (n=3).
