# Supplementary material for: In vivo acute toxicity evaluation and in vitro molecular mechanism study of antiproliferative activity of a novel indole Schiff base β-diiminato manganeseIII complex in hormone-dependent and triple negative breast cancer cells
Source: PeerJ. 2019 Oct 7;7:e7686. doi: 10.7717/peerj.7686 (PMC6786247; doi:10.7717/peerj.7686)
Supplement: Supplemental Information 3 [file peerj-07-7686-s003.docx]

**MCF-7**

**Untreated control**

**
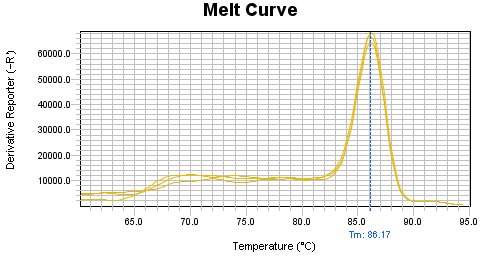
GAPDH**


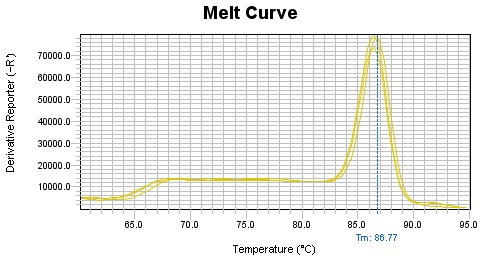


**BAX**


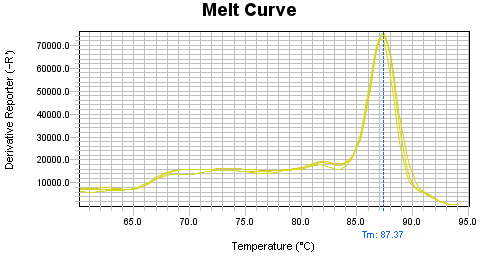


**BCL-2**


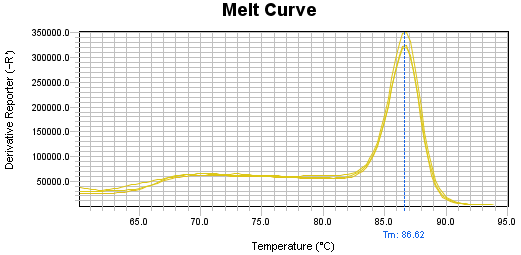


**P21**


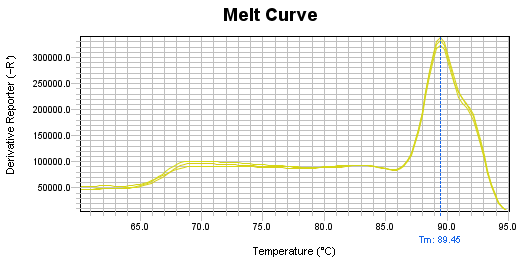


**Cyclin D1**


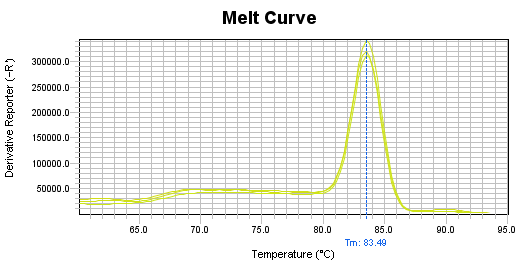


**Caspase-3**


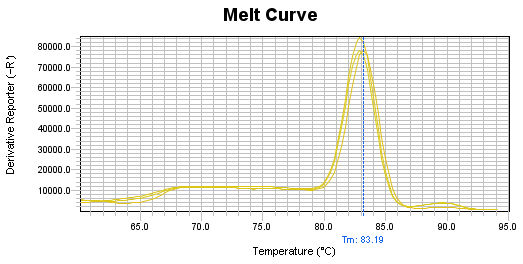


**Caspase-8**


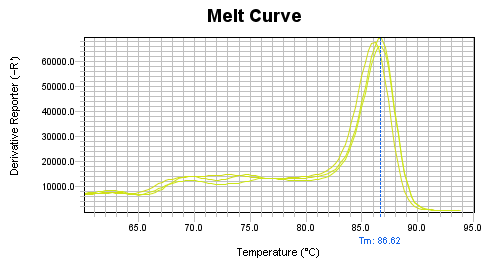


**Caspase-10**


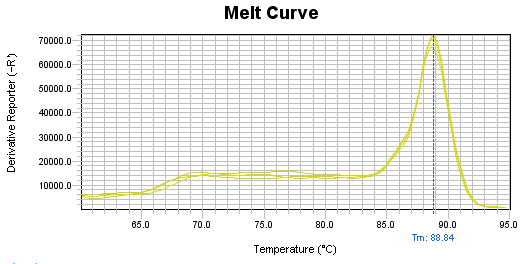


**TNF-α**


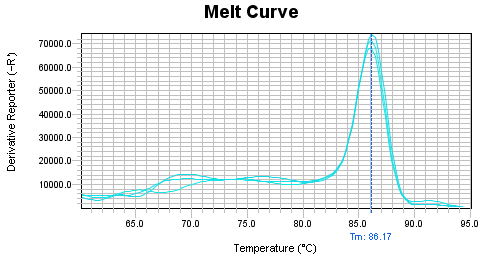
**Mn^III^ complex**


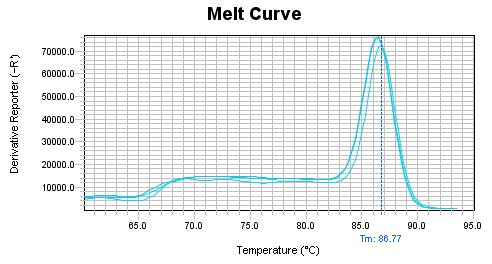
**GAPDH**

**BAX**


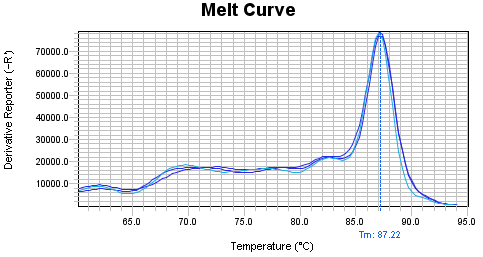


**BCL-2**


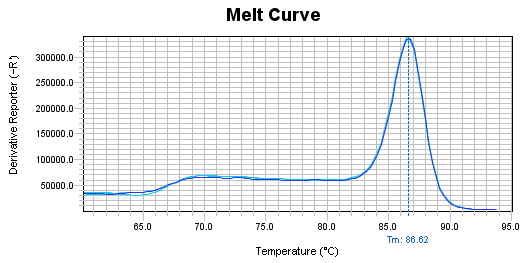


**P21**


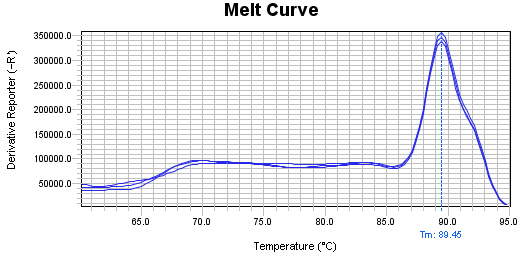


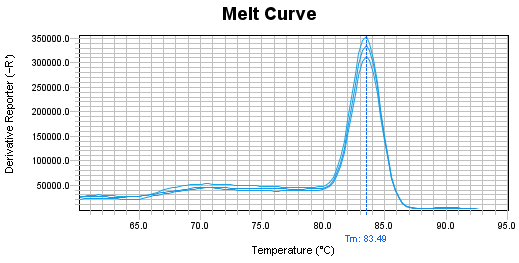
**Cyclin D1**

**Caspase-3**


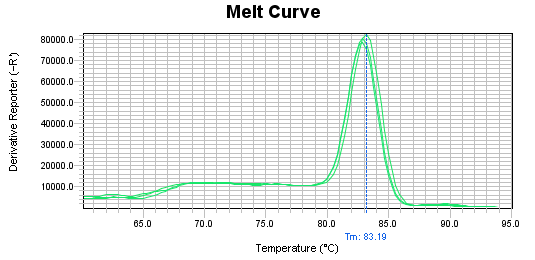


**Caspase-8**


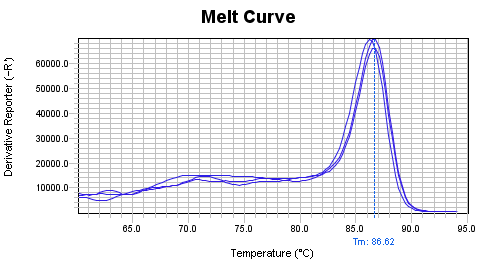


**Caspase-10**


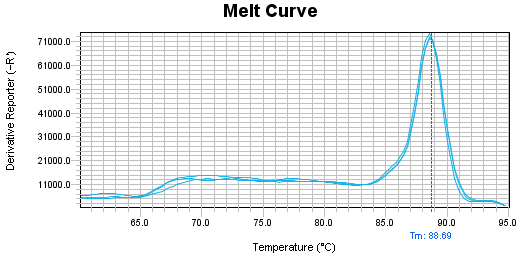
**TNF-α**

**MDA-MB-231**

**Untreated control**


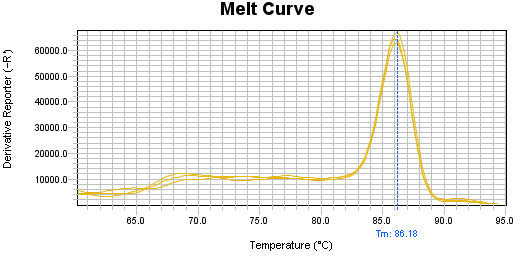
**GAPDH**


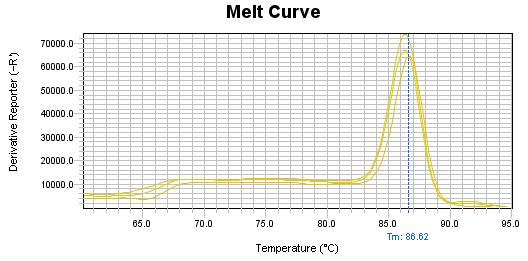


**BAX**


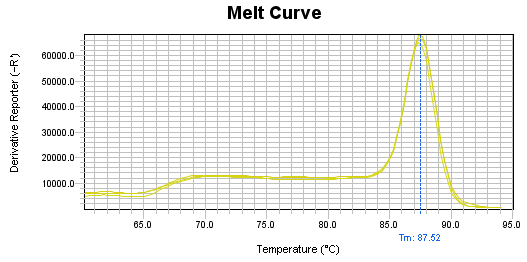


**BCL-2**


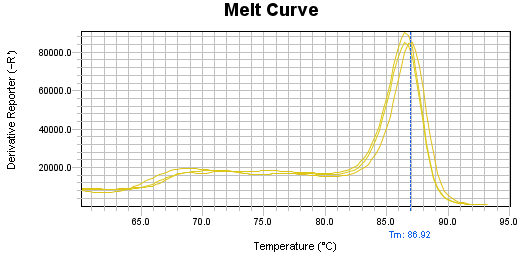


**P21**


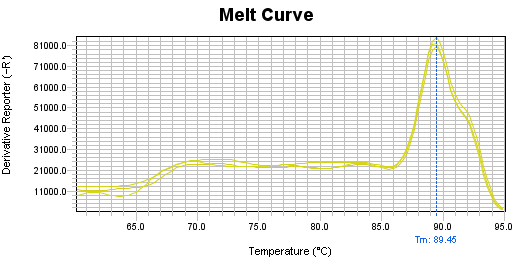


**Cyclin D1**


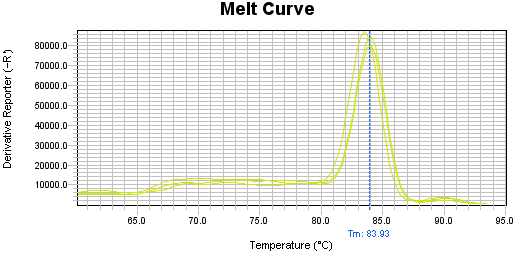


**Caspase-3**

**
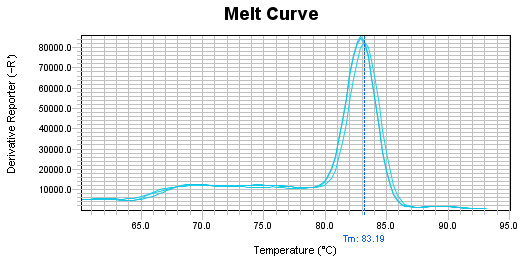
Caspase-8**


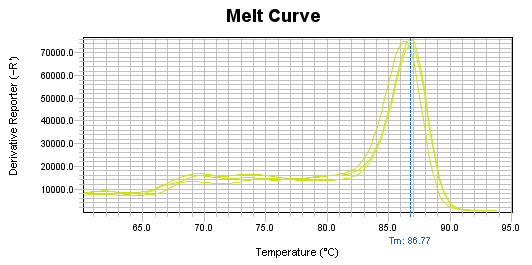


**Caspase-10**


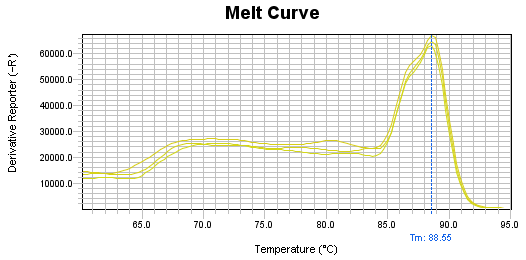


**TNF-α**

**Mn^III^ complex**


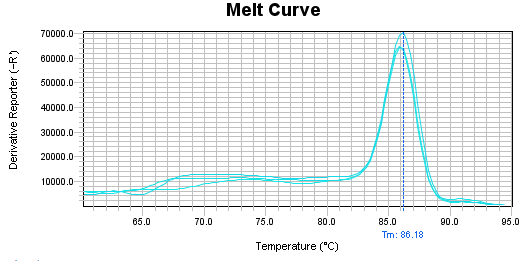
**GAPDH**


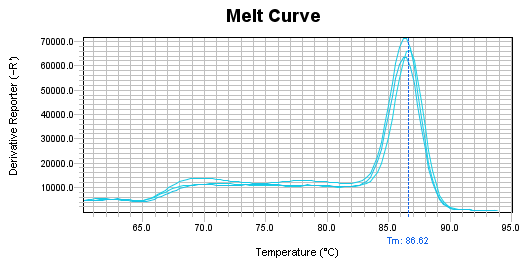


**BAX**


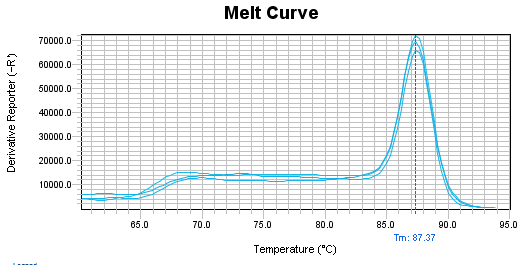


**BCL-2**


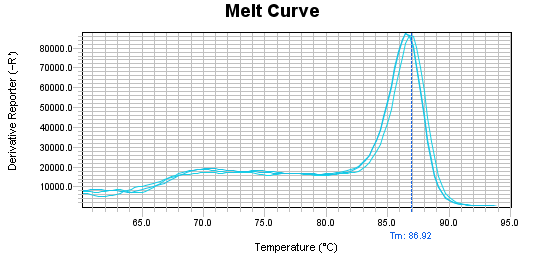


**P21**


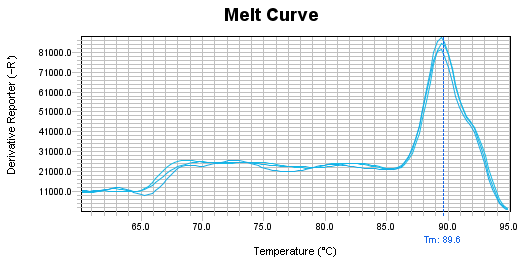


**Cyclin D1**


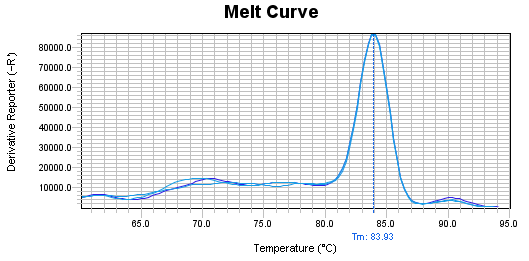


**Caspase-3**

**
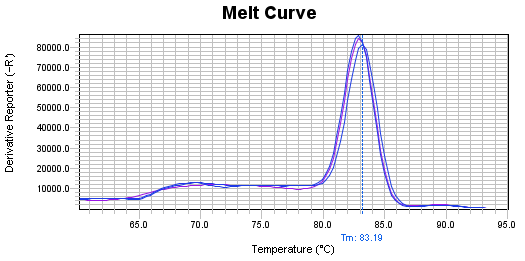
Caspase-8**


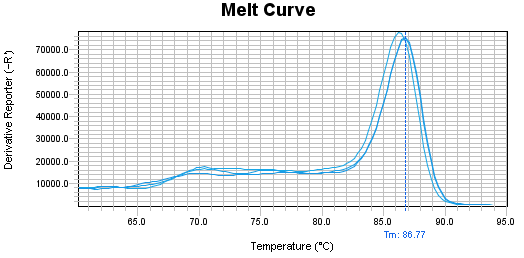


**Caspase-10**


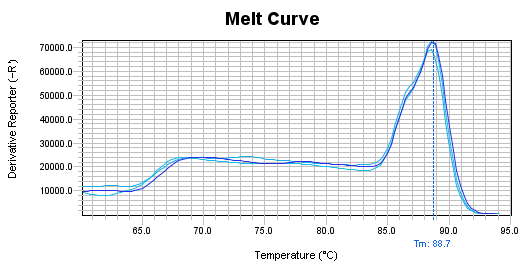


**TNF-α**
